# Pollen-mediated transfer of herbicide resistance between johnsongrass (*Sorghum halepense*) biotypes

**DOI:** 10.1038/s41598-022-11713-8

**Published:** 2022-05-10

**Authors:** Aniruddha Maity, Blake Young, Nithya Subramanian, Muthukumar Bagavathiannan

**Affiliations:** Department of Soil and Crop Sciences, Texas A&M University, College Station, TX USA

**Keywords:** Ecology, Evolution, Plant sciences

## Abstract

Johnsongrass (*Sorghum halepense*) is a troublesome weed in row crop production in the United States. Herbicide resistance is a growing concern in this species, with resistance to ACCase-, ALS-, and EPSPS-inhibitors already reported. Pollen-mediated gene flow (PMGF) is capable of spreading herbicide resistance, but the extent of PMGF has not yet been studied in johnsongrass. Field experiments were conducted in a Nelder-wheel design to quantify the distance and frequency of PMGF from ALS-inhibitor-resistant (AR) to -susceptible (AS) johnsongrass across three environments (summer 2018, fall 2018, and fall 2019). The AR biotype (pollen donor) was established at the center of the wheel (5-m diameter), and a naturally occurring johnsongrass (AS) infestation was utilized as the pollen recipient, in eight directions and at nine distances (5, 10, 15, 20, 25, 35, 40, 45, and 50 m) within each direction. Seeds collected from the AS plants in each distance and direction were screened for survival to the ALS-inhibitor herbicide nicosulfuron (Accent Q) at 95 g ai ha^−1^ under greenhouse conditions. The survivors (i.e. hybrids) were further confirmed based on the presence of the Trp_574_Leu mutation. At the closest distance of 5 m, PMGF was 9.6–16.2% across the directions and environments, which progressively declined to 0.8–1.2% at 50 m. The exponential decay model predicted 50% reduction in PMGF at 2.2 m and 90% reduction at 5.8 m from the pollen donor block. Results demonstrate that herbicide resistance can spread between adjacent field populations of johnsongrass through PMGF, which necessitates sound monitoring and management.

## Introduction

Johnsongrass [*Sorghum halepense*) (L.) Pers.] (2n = 4X = 40) is believed to be a natural hybrid between *S. bicolor* (2n = 2X = 20), an annual species native to eastern Africa, and *S. propinquum* (2n = 2X = 20), a perennial species native to Southeast Asia^1–3^. Although johnsongrass has been used as fodder and human food (grain/flour) in many countries, it is primarily known as a troublesome weed across the globe^4^. As a perennial weed species, johnsongrass is capable of invading and persisting in a wide range of habitats, from minimally disturbed ruderal areas to cultivated fields^4–6^. The presence of high phenotypic plasticity, genetic diversity, stress tolerance, potential production of allelochemicals, and association with nitrogen-fixing bacterial endophytes make it a successful weed species under diverse environments (ENVs)^7–10^.

Johnsongrass was introduced into the United States (U.S.) approximately 200–300 years ago as a promising perennial forage species, but later became one of the most noxious and invasive agricultural weeds^4,6^. This species is now listed as a noxious weed in 20 U.S. states and an invasive species in 16 states^11^. Johnsongrass severely impacts summer row crops such as grain sorghum, cotton, corn, and soybean in the U.S.^12,13^, driving significant annual spending for its management^14^. Management of this species is particularly challenging in grain sorghum because of the genetic similarities between the two species, and the lack of control options using selective herbicides^15^.

In addition to vegetative reproduction through the extensively creeping underground rhizomes of about 40–90 m per plant^16^, a single johnsongrass plant can produce as many as 28,000 seeds through sexual reproduction^17^. Enormous seed production followed by their shattering aids in spatial seed dispersal and seedbank enrichment. This assists in johnsongrass species persistence by overcoming the agronomic management practices or natural calamities^9,18^. Sexual reproduction also equips the species to successfully transfer/introgress alleles into new genetic backgrounds, improving adaptive potential under new ENVs and ultimately contributing to overall species persistence^19,20^.

Johnsongrass exhibits prolonged flowering during the growing season due to continuous production of tillers, which allows for flowering synchrony between different johnsongrass populations, and also with other compatible *Sorghum* spp. in agricultural landscapes^21–23^. The flowers of johnsongrass are bisexual and chasmogamous, with an andromonoecious sexual system favoring self-pollination^24^. However, the precocious extrusion of receptive stigmas preceding anther dehiscence, availability and flow of abundant pollen of high fertility and longevity, and high seed setting rate lead to substantial rates of outcrossing in johnsongrass^25^. Pollen of the members of *Sorghum* genera can travel long distances^8^. However, to the best of our knowledge, no detailed report is available on gene flow between johnsongrass populations. In related species, cross-fertilization (between sorghum and johnsongrass) is not reported at distances greater than 130 m^26^. Arriola and Ellstrand^27^ documented as high as 2% outcrossing in johnsongrass (tetraploid) pollinated by sorghum (diploid) pollen at a distance of 100 m, whereas Schmidt et al.^28^ reported as high as 2.6% hybridization in shattercane [*S. bicolor* nothosubsp. drummondii (Steud.) de Wet ex Davidse] at 200 m distance from sorghum plants. It is likely that the rate of pollen-mediated gene flow (PMGF) between johnsongrass populations could be greater at these distances due to a lack of ploidy differences. This may have immense implications for pollen-mediated flow of herbicide resistance gene(s) at both intra- and interspecific levels^27,29^.

Johnsongrass is also one of the high-risk species for evolving resistance to herbicides. So far, resistance to four different herbicide modes of action (MOAs) [acetolactate synthase (ALS)-, acetyl CoA carboxylase (ACCase)-, enolpyruvylshikimate-3-phosphate synthase (EPSPS)-, and microtubule assembly-inhibitors] has been reported across nine U.S. states^30^. There have also been reports of populations showing multiple resistance to ALS-, ACCase-, and EPSPS-inhibitors^14,31–33^. The upcoming or newly commercialized herbicide-resistant sorghum technologies (e.g. ALS-inhibitor resistance) may impose additional selection pressure for rapid herbicide resistance evolution in johnsongrass through the selective use of certain herbicides. Moreover, the potential for hybridization between herbicide-resistant sorghum and johnsongrass may introduce the resistance trait in this species^27,34,35^, which may further be transferred to other susceptible johnsongrass biotypes through gene flow. In this regard, developing an understanding of gene flow and the potential for the spread of novel traits between johnsongrass populations is imperative. Gene flow between johnsongrass populations can occur through the movement of vegetative propagules, pollen, and/or seed. The current research focuses on PMGF.

Potential for PMGF among the members of the genus *Sorghum* is reviewed by Ohadi et al.^15^. Existing reports specifically address PMGF from cultivated sorghum to johnsongrass^27^; however, there is a lack of knowledge on the rate of PMGF between johnsongrass populations. The extent of PMGF is influenced by various factors such as plant biology, ENV conditions, and spatial and temporal separation of the pollen donor and the recipient. Ellstrand^36^ suggested that the extent of PMGF may be asymmetrical in response to temporal and spatial variations, thus multi-ENV field experiments are vital for determining practical PMGF frequencies.

Herbicide resistance conferred by target-site mutations can be reliably detected using the phenotypic response combined with molecular markers, and has been widely used in studies investigating the frequency of intra-specific gene flow between populations^37,38^. The ALS-inhibitor resistance in johnsongrass is typically controlled by specific, detectable mutation(s) and provides a unique opportunity to identify the recipient that carries the resistance-conferring allele^39^. Here, a multi-ENV experiment was conducted using a Nelder wheel design (multi-directional arrangement) to assess PMGF in johnsongrass. In this regard, a known ALS-inhibitor-resistant (AR) johnsongrass biotype was used as the pollen donor and a susceptible (AS) johnsongrass biotype was used as the pollen recipient. The rates of PMGF were determined by using the herbicide resistance trait as the trackable marker. Hybrid plants were confirmed phenotypically and using a molecular marker specific to the resistance-conferring mutation.

## Materials and methods

### Plant materials

An ALS-inhibitor-resistant johnsongrass (resistant to nicosulfuron) obtained from the University of Nebraska-Lincoln (source credit: Dr. John Lindquist) was used as the pollen source (male parent), and the natural johnsongrass population present in the experimental field at the Texas A&M University Farm, Somerville (Burleson County), Texas (30° 32′ 15.4″ N 96° 25′ 49.2″ W) with no history of ALS-inhibitor resistance was used as the pollen recipient (female parent). Prior to the initiation of the field experiment, the susceptibility to nicosulfuron of the natural johnsongrass population was verified by spraying Accent Q at the labeled field rate of 63 g ai ha^−1^ [mixed with 0.25% v/v Crop Oil Concentrate (COC)] on 10 randomly selected 1 m^2^ johnsongrass patches across the field area at 15–30 cm tall seedling stage. For this purpose, a CO_2_ pressurized backpack sprayer was calibrated to deliver 140 L ha^−1^ of spray volume at an operating speed of 4.8 kmph. The natural johnsongrass population was determined to be completely susceptible to nicosulfuron.

During spring 2018, the seeds of AR johnsongrass were planted in pots (14-cm diameter and 12-cm tall) filled with potting soil mixture (LC1 Potting Mix, Sungro Horticulture Inc., Agawam, MA, USA) at the Norman Borlaug Center for Southern Crop Improvement Greenhouse Research Facility at Texas A&M University. The environmental conditions were set at 26/22 °C day/night temperature regime and a 14-h photoperiod. In each pot, 5 seeds were planted and thinned to one healthy seedling at 1-leaf stage. Seedlings were supplied with sufficient water and nutrients (Miracle-Gro Water Soluble All Purpose Plant Food, Scotts Miracle-Gro Products Inc., 14111 Scottslawn Road, Marysville, OH 43041). A total of 50 seedlings were established in the greenhouse and were maintained until they reached about 10 cm tall, at which point they were sprayed with 2× the field rate of nicosulfuron (63 × 2 = 126 g ai ha^−1^) (mixed with 0.25% v/v COC). The herbicide was applied using a track-sprayer (Research Track Sprayer, DeVries, Hollandale, MN) fitted with a flat fan nozzle (TeeJet XR110015) that was calibrated to deliver a spray volume of 140 L ha^−1^ at 276 kPa pressure, and at an operating speed of 4.8 kmph. All treated seedlings that survived the herbicide application at 21 days after treatment (DAT) were then used as the pollen donor in the field gene flow experiment. All plant materials were handled in accordance with relevant guidelines and regulations. No permissions or licenses were required for collecting the johnsongrass samples from the experimental fields.

### Dose–response assays

The degree of resistance/susceptibility to nicosulfuron of the AR and AS johnsongrass biotypes were determined using a classical dose–response experiment. The assay consisted of seven rates (0, 0.0625, 0.125, 0.25, 0.5, 1, and 2×) for the AS population and nine rates (0, 0.25, 0.5, 1, 2, 4, 8, 16, and 32×) for the AR population [1 × (field recommended rate) = 63 g ai ha^−1^ of Accent Q]. The experimental units were arranged in a completely randomized design with four replications. Seeds of AR and AS plants were planted in plastic trays (25 × 25 cm) filled with commercial potting-soil mix (LC1 Potting Mix, Sungro Horticulture Inc., Agawam, MA, USA) and maintained at 26/22 °C day/night cycle with a 14-h photoperiod in the greenhouse. Seedlings at 1–2 leaf stage were thinned to 20 seedlings per tray; four replications each of 20 seedlings per dose were considered. The seedlings were watered and fertilized as needed. The assay was conducted twice, thus a total of 160 seedlings were screened for each dose.

The established seedlings were sprayed with the appropriate herbicide dose at the 10–15 cm tall seedling stage. The herbicide was applied using a track sprayer calibrated to deliver a spray volume of 140 L ha^−1^ at 4.8 kmph operating speed. Survival (%) and injury (%) were assessed at 28 DAT. Any plant that failed to grow out of the herbicide impact was considered dead. Plant injury was rated for each plot (i.e. on the 20 seedlings per rep) on a scale of 0–100%, where 0 indicates no visible impact compared to the nontreated control and 100 indicates complete death of all plants in the tray. Immediately after the visual ratings were completed, shoot biomass produced by the 20 plants from each tray was determined by harvesting all the tissues at the soil level and drying them in an oven at 60 °C for 72 h. Seedling mortality data were used for fitting dose–response curves that allowed for determining the lethal dose that caused 100% mortality of the susceptible biotype. This dose was used as a discriminant dose to distinguish between a hybrid (that confers resistance to nicosulfuron as a result of gene flow) and a selfed progeny (susceptible to nicosulfuron) in the field gene flow study.

### Field experimental location and set-up

**Figure 1:**
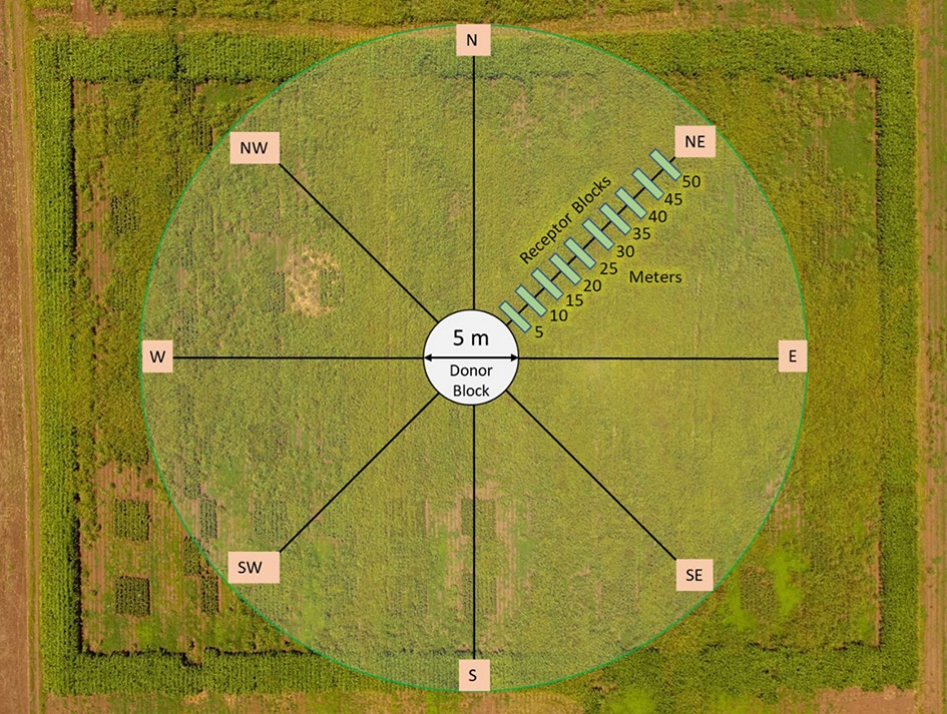
Aerial view of the experimental arrangement that was used to quantify pollen-mediated gene flow from ALS-inhibitor resistant (AR) to -susceptible (AS) johnsongrass at the Texas A&M University Research Farm near College Station, Texas. AR johnsongrass plants were transplanted in the pollen-donor block of 5 m diameter at the center of the field. The surrounding pollen-receptor area was divided into four cardinal (N, E, S, W) and four ordinal (NE, SE, SW, NW) directional blocks where naturally-existing AS johnsongrass plants were used as the pollen-recipients. AS panicles exhibiting flowering synchrony with AR plants were tagged at specific distances (5–50 m, at 5 m increments) along the eight directional arms. A tall-growing biomass sorghum border was established in the perimeter of the experimental site to prevent pollen inflow from outside areas.

The field experiment was conducted across two ENVs in 2018 (summer and fall) and one in 2019 (fall) at the Texas A&M University Farm, Somerville (Burleson County), Texas (30° 32′ 15.4″ N 96° 25′ 49.2″ W). The study site is characterized by silty clay loam soil with an average annual rainfall of 98.2 cm. The field experiment followed the Nelder-wheel design^40^, i.e. concentric donor-receptor design, a widely used method for gene flow studies, wherein the pollen-donors are surrounded by the pollen-receptors (Fig. 1). In this study, the AR plants (planted in the central block of the wheel) served as the pollen-donors, whereas the AS plants (present in the spokes) served as the pollen-receptors.

The center of the wheel was 5 m in diameter, and each spoke was 50 m long starting at the periphery of the central circular block. Thirty AR plants (pollen-donors) were transplanted in four concentric rings of 1, 5, 9, and 15 plants in the 5 m diameter central block, surrounded by the pollen-receptors (i.e. AS plants) (Fig. 1). The AR plants were contained within the central block during the 2 years of the field experiment by harvesting and removing all mature seeds and removing any expanding rhizomatous shoots. Further, field cultivation was completely avoided in the central block throughout the study period. Any newly emerging johnsongrass plants (seedling/rhizomatous) other than the transplanted AR plants in the central block were removed periodically by manual uprooting.

The wheel consisted of eight spokes (i.e. directional blocks) arranged in four cardinal (N, E, S, W) and four ordinal (NE, SE, NW, SW) directions (Fig. 1). The plots to quantify gene flow frequency were arranged at 0 (border of the central block), 5, 10, 15, 20, 25, 30, 35, 40, 45, and 50 m distances from the central block in all eight directions (Fig. 1). Each plot measured 3 × 2 m and the area surrounding the plots was shredded prior to the booting stage with a Rhino^®^ RC flail shredder (RHINOAG, INC., Gibson City, IL 60936).

A tall-growing biomass sorghum border (6 m wide) was established surrounding the experimental area in all directions to prevent potential inflow of pollen from other *Sorghum* spp. in the nearby areas. Additionally, prevailing weather conditions, specifically wind direction, wind speed, relative humidity, and air temperature measured at 5-min intervals were obtained from a nearby weather station located within the Texas A&M research farm (http://afs102.tamu.edu/). The field did not require any specific agronomic management in terms of irrigation, fertilization, or pest management.

### Flowering synchrony, tagging, and seed harvesting

At peak flowering, when > 50% of the plants in the AR block started anther dehiscence (i.e., pollen shedding), ten AS panicles (five random plants × 2 panicles per plant) that showed flowering synchrony with AR plants and displayed protruded, receptive stigma were tagged using colored ribbons at each distance and direction. At seed maturity, the tagged AS panicles were harvested separately for each distance and direction. Panicles were threshed, seeds were cleaned manually, and stored under room conditions until used in the herbicide resistance screening to facilitate after-ripening and dormancy release.

### Resistance screening

The hybrid progeny produced on AS plants as a result of outcrossing with AR plants would be heterozygous for the allele harboring nicosulfuron resistance, and would exhibit survival upon exposure to the herbicide applied at the discriminant dose at which all wild type (AS) plants would die. The discriminant dose was determined using the dose–response study described above. Thus, the frequency of resistant plants in the progeny would represent outcrossing/gene flow (%).

To effectively detect the levels of gene flow from AR to AS biotypes especially at low frequencies, the minimum sample size required for resistance screening was determined based on the following formula (Eq. 1)^41^:

1 $${\text{N }} = {\text{ ln}}\left( {{1} - P} \right)/{\text{ln}}\left( {{1} - p} \right),$$

where *P* is the probability of detecting a resistant progeny in the least frequent class and *p* is the probability of the least frequent class. Based on this formula, a minimum of 298 to as high as 916 plants were screened for each distance within each direction, allowing for a 1% detection level (*p* = 0.01) with a 95% (*P* = 0.95) confidence interval.

Approximately one-year old progeny seeds harvested from the AS plants were scarified using a sandpaper for 15–20 s to release dormancy. The seeds for each distance within each direction were planted in four replicates of plastic trays (50 × 25 cm) filled with potting soil mixture (LC1 Potting Mix, Sungro Horticulture Inc., Agawam, MA, USA). The plants were raised at the Norman Borlaug Center for Southern Crop Improvement Greenhouse Research Facility at Texas A&M University. The greenhouse was maintained at 28/24 °C day/night temperature regime and a 14-h photoperiod. About 10–15 cm tall seedlings were sprayed with the discriminant dose of the ALS-inhibitor nicosulfuron (Accent Q, 95 g ai ha^−1^) using a spray chamber (Research Track Sprayer, DeVries, Hollandale, MN) fitted with a flat fan nozzle (TeeJet XR110015) that was calibrated to deliver a spray volume of 140 L ha^−1^ at 276 kPa pressure, operating at a speed of 4.8 kmph. At 28 DAT, percent seedling survival was determined based on the number of plants that survived the herbicide application out of the total number of plants sprayed. The number of plants in each tray was counted before spraying.

### Molecular confirmation of hybrids

Leaf tissue samples were collected from thirty random surviving plants (putative resistant) in the herbicide resistance screening study for each of the three field ENVs, thus totaling 90 samples. Genomic DNA was extracted from 100 mg of young leaf tissue using the modified CTAB protocol^42^. The concentration of DNA was determined using a Nanodrop 1000 UV–Vis spectrophotometer (DeNovix DS-II spectrophotometer, DeNovix Inc., Wilmington, DE 19810, USA). DNA was then diluted to a concentration of 20 ng/µl for PCR assay. The nicosulfuron-resistant johnsongrass from Nebraska used in this study possessed the Trp_574_Leu mutation^39^. Hence, single nucleotide polymorphism (SNP) primers targeting a unique short-range haplotype of Inzen^®^ sorghum (Val_560_Ile + Trp_574_Leu) were performed using the PCR Allele Competitive Extension (PACE) platform to confirm the resistant plants^43^. The SNP primers and the PACE genotyping master mix were obtained from Integrated DNA Technologies (IDT) Inc. (Coralville, IA) and 3CR Bioscience (Harlow CM20 2BU, United Kingdom), respectively. In addition to the two no-template controls (NTCs), two nicosulfuron-resistant johnsongrass, one wild-type johnsongrass, and one Inzen^®^ sorghum were also used in the PCR.

The PCR was performed according to the manufacturer’s protocol (Bio-Rad Laboratories, Inc., Hercules, CA), with denaturation for 15 min at 94 °C, followed by 10 cycles of denaturation at 94 °C for 20 s, annealing and extension at 65–57 °C for 60 s, 30 cycles of denaturation for 20 s at 94 °C, and annealing and extension for 60 s at 57 °C. Fluorescence of the reaction products were detected using a BMG PHERAStar plate reader that uses the FAM (fluorescein amidite) and HEX (hexachloro-fluorescein) fluorophores.

### Data analysis

For the dose–response assay, three-parameter sigmoidal curves (Eq. 2) were fit on the seedling mortality data for the AS and AR biotypes (with log of herbicide doses), using SigmaPlot version 14.0 (Systat Software Inc., San Jose, CA).

2 $$y=b/[1+{exp}^{\left(-(x-e\right)/c)}],$$

where, *y* is the mortality (%), *x* is the herbicide dose (g ai ha^−1^), *b* is the slope around *e*, *c* is the lower limit (theoretical minimum for *y* normalized to 0%), and e = LD_50_ (inflection point, mid-point or estimated herbicide dose when *y* = 50%). Windrose plots that represented wind speed and frequency during the flowering window in each of the eight directions were created using a macro in Microsoft Excel. Progeny seedling survival (%) that represents gene flow (%) was determined using Eq. (3).

3 $${\text{PMGF }}\left( {\text{\%}} \right){ } = { }\left( \frac{X}{Y} \right)_{{i,j{ }}} \times { }100,$$

where, *X* is the number of plants that survived the herbicide application, *Y* is the total number of plants sprayed for *i*th distance in *j*th direction.

To test whether gene flow frequencies varied among the directions, ANOVA was conducted using JMP PRO v.14 (SAS Institute, Cary, NC, USA), based on the average gene flow frequency values in each direction; ENVs were considered as replicates in this analysis. A non-linear regression analysis for gene flow rate, describing an exponential decay function (Eq. 4), was fit using SigmaPlot based on the gene flow frequencies observed at different distances pooled across the directions and ENVs.

4 $$y=y0+\left[a\times {exp}^{\left(-b\times x\right)}\right],$$

where, *y* is the PMGF (%), *x* is the distance (m) from pollen source, *y0* is the lower asymptote (theoretical minimum for *y* normalized to 0%), *a* is the inflection point, mid-point or estimated distance when *y* = 50%, and *b* is the slope around *a*.

A Pearson correlation analysis was conducted to determine potential association between PMGF [overall PMGF, short-distance PMGF (5 m), and long-distance PMGF (50 m)] and the environmental parameters temperature, relative humidity, and dew point. Further, a correlation analysis was also conducted to understand the association between PMGF frequencies and specific wind parameters such as wind frequency, wind speed, and gust speed. The molecular data were analyzed using KlusterCaller 1.1 software (KBioscience).

## Results and discussion

### Dose response

**Figure 2:**
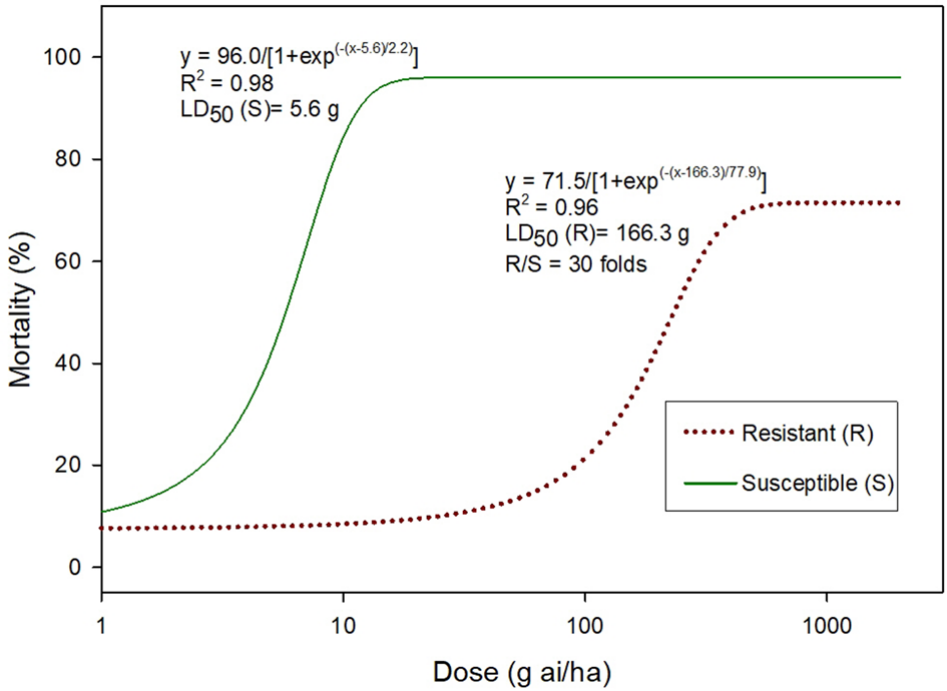
Dose–response for the ALS-inhibitor nicosulfuron (Accent Q) herbicide resistant (pollen donor) and susceptible (pollen receptor) johnsongrass plants used in the study. The susceptible plants were sprayed at seven doses (0, 0.0625, 0.125, 0.25, 0.5, 1, and 2×) and the resistant plants were sprayed at nine doses (0, 0.25, 0.5, 1, 2, 4, 8, 16, and 32×) (1× = 63.05 g ai/ha). Plant survival was evaluated at 28 days after herbicide application. In the equation, y = mortality (%), x = herbicide dose (g ai/ha); LD_50_ = amount of herbicide that killed 50% of the test plants; R/S = LD_50_ of R biotype/LD_50_ of S biotype.

**Figure 3:**
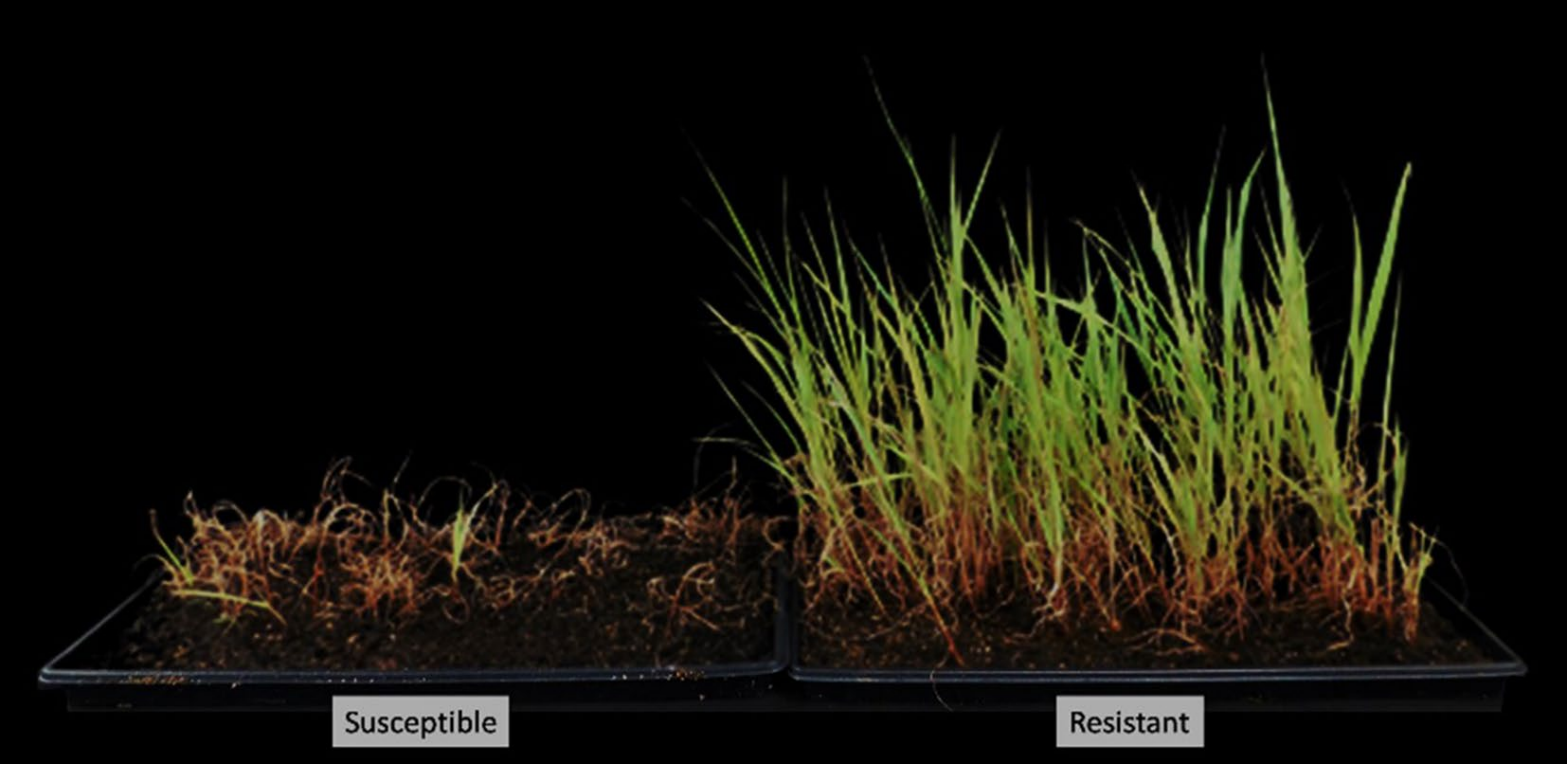
The response of susceptible (AS) and resistant (AR) plants at the discriminant rate of the ALS-inhibitor nicosulfuron (Accent Q, 63 g ai/ha).

The dose–response analysis showed that the AS biotype (i.e. susceptible johnsongrass) did not survive the field rate (1×, 63 g ai ha^−1^) of nicosulfuron; the majority of AS plants died at the 0.5 × rate (Fig. 2). However, the AR biotype showed strong resistance to nicosulfuron, with survival up to the 32 × rate, the highest rate tested here (Fig. 3). The LD_50_ values were 5.6 and 166.3 g ai ha^−1^, respectively for the AS and AR biotypes, with an R/S ratio of 30. Based on the dose–response assay, a discriminant dose of 95 g ai ha^−1^ (1.5×) was used to select hybrid progenies that contained the resistance-conferring mutation. At this dose, the heterozygote hybrid progeny is expected to survive the herbicide, whereas none of the susceptible wild-type progeny would survive. Werle et al.^44^ tested the resistance level of putative ALS-inhibitor resistant johnsongrass biotypes collected from Nebraska and Kansas and reported a 4.9 (J-35) and > 1000-fold resistance (J-36) in two resistant johnsongrass biotypes, which required 0.61 and 446.4 g ai ha^−1^ nicosulfuron, respectively to achieve 50% growth reduction. The biotype used here had a relatively lower level of resistance than that of J-36, yet was sufficient to effectively discriminate between the AS and AR biotypes. Thus, the AR biotype was an appropriate genotype for utilization in this gene flow study.

### Flowering synchrony

Naturally occurring johnsongrass plants in the experimental area showed reproductive maturity at least twice within a growing season; the first flowering began in May that lasted through August, followed by a second flush from September through October. Regular monitoring and documentation of flowering phenology in the central as well as directional blocks confirmed high levels of flowering synchrony between the pollen donors (AR plants) and receptors (AS plants); thus, selection and tagging of the individual AS panicles (that showed flowering synchrony with AR plants) for seed collection were easy. The profuse tillering habit and indeterminant growth nature of johnsongrass allowed for high levels of flowering synchrony.

Johnsongrass exhibits a wide flowering window that coincides with the flowering times of other *Sorghum* spp. such as shattercane and grain sorghum^15,21–23^. Wide flowering windows in johnsongrass were also reported in California^45^, Kansas, Missouri, and Nebraska^44^, which corroborates our observations. The high degree of flowering synchrony allows for natural outcrossing among members of *Sorghum* genera and accelerates the dispersal of resistance-conferring alleles and other novel traits^3,9,15,46^.

### Frequency of PMGF

**Figure 4:**
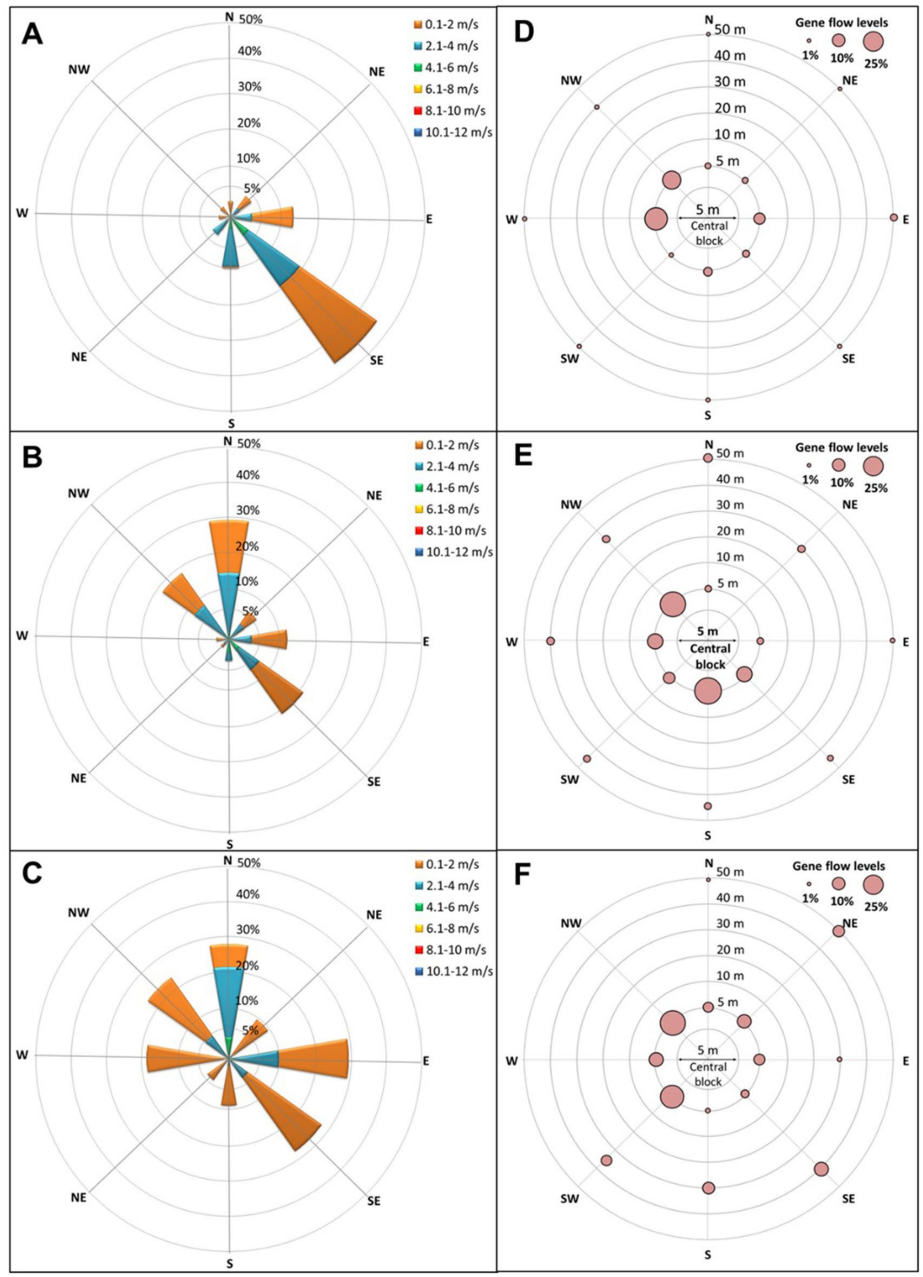
(**A**–**C**) Windrose plots illustrating the speed (m/s) and frequency (%) of wind events in the four cardinal (N, E, S, W) and four ordinal (NE, SE, SW, NW) directions at the experimental site during johnsongrass flowering in (**A**) Environment 1, Jun–July, 2018; (**B**) Environment 2, October–November, 2018; and (**C**) Environment 3, August–September, 2019. Wind speed was grouped into various categories, depending on the overall wind speed recorded during the observation period. (**D**–**F**) Gene flow (%) at the closest (5 m) and the farthest (50 m) distance of detection in all eight directions for the environments corresponding with (**A**) to (**C**). The size of the circles provides a reference for gene flow level.

**Figure 5:**
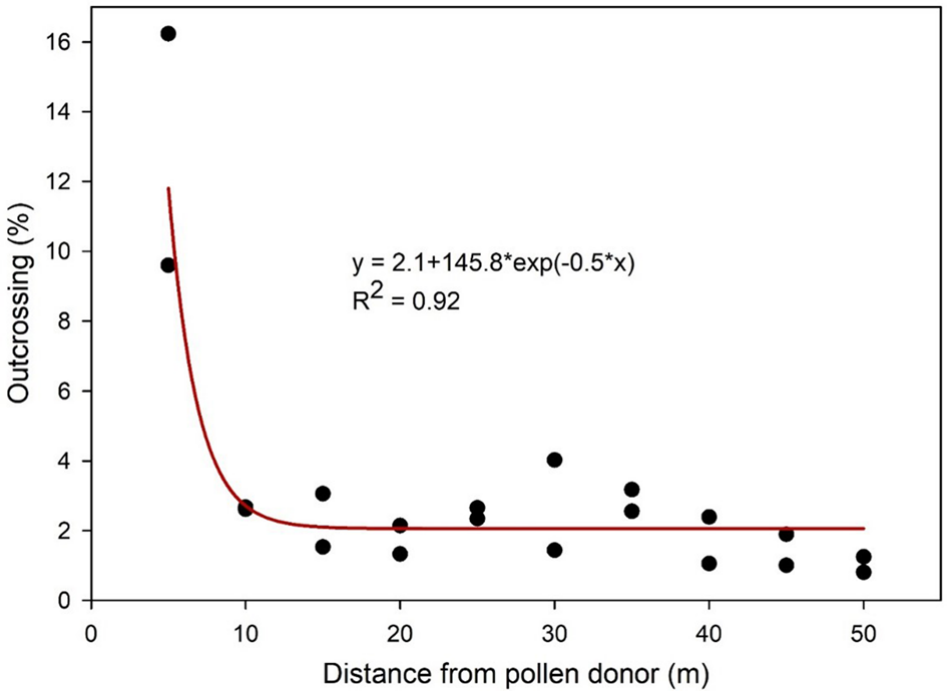
An exponential decay curve [f = y0 + a × exp(− b × x)] showing mean outcrossing (y) as a function of distance (x) from the pollen source, averaged among all directions across three study environments: Environment 1, Jun–July, 2018; Environment 2, October–November, 2018; and Environment 3, August–September, 2019.

The PMGF frequency, determined as the frequency of survivors to the discriminant dose of nicosulfuron in the progeny harvested from the AS plants, was considerably high and varied among the directions and the ENVs (Supplementary Table S1 and Fig. 4). At the 5 m distance, PMGF ranged from 0 to 45% across the directions and ENVs. The average PMGF (averaged across directions within an Env) at the 5 m distance varied from 9.6 to 16.2% across the three ENVs, which progressively declined with the increasing distance from the AR block (Table 1). At 50 m, the farthest distance investigated here, the average PMGF ranged from 0.8 to 1.2% across the ENVs; however, it could be as high as 8.7% in a specific direction in a particular ENV (Supplementary Table S1). The windrose plots describe the frequency and speed of wind events in different directions (Fig. 4A–C). A wheel diagram demarcated with the gene flow (%) in each direction is presented (Fig. 4D–F), and the entire gene flow dataset for each direction and distance for the three ENVs is provided in the Supplementary Table S1. The exponential decay function developed based on PMGF recorded at each increasing distance from the pollen source across the directions and ENVs is shown in Fig. 5. The model predicted 50% reduction in PMGF at 2.2 m and 90% reduction at 5.8 m from the pollen donor block.

**Table 1:** Frequency of pollen-mediated gene flow in johnsongrass for distances up to 50 m from the pollen source in three environments.

Distance (m)	Environment 1			Environment 2			Environment 3		
	Plants screened	Plants survived	Gene flow (%)	Plants screened	Plants survived	Gene flow (%)	Plants screened	Plants survived	Gene flow (%)
5	3487	335	9.6	925	150	16.2	997	145	14.5
10	2368	63	2.7	1032	27	2.6	939	28	3.0
15	2769	85	3.1	859	13	1.5	820	14	1.7
20	3445	74	2.1	1214	16	1.3	1100	0	0.0
25	3595	84	2.3	1067	28	2.7	796	16	2.0
30	2518	36	1.4	1089	44	4.0	1159	19	1.6
35	3252	83	2.6	958	30	3.2	1084	37	3.4
40	2516	60	2.4	889	9	1.1	1087	23	2.1
45	2267	43	1.9	1343	13	1.0	1667	16	0.9
50	2525	31	1.2	466	4	0.8	655	7	1.1
Total	28,742	895	2.9	9842	336	3.4	10,304	303	3.0

Johnsongrass seed collection time: Environment 1, July 2018; Environment 2, November 2018; and Environment 3, September 2019.

The current study provides a detailed account of hybridization potential between johnsongrass biotypes across multiple ENVs. We used percent hybridization as a measure of PMGF because gene flow should include successful integration of the trait in the progeny^47^. While hybridization between sorghum and johnsongrass was studied earlier^48,49^, to our knowledge this is the first study that investigated PMGF between two johnsongrass biotypes. The study by Arriola and Ellstrand^27^ reported an outcrossing frequency of up to 2% in johnsongrass at 100 m distance from sorghum; it is notable that ploidy difference between sorghum (2*n* = 2*X* = 20) and johnsongrass (2*n* = 4*X* = 40) is expected to reduce outcrossing frequency between the two species^34^. Considerable outcrossing frequencies documented in the current study at 50 m distance is not surprising, especially given that there are no ploidy barriers between johnsongrass biotypes.

Though the distance of pollen movement has not been studied in johnsongrass, grass pollen is typically known to travel for long distances, often facilitated by wind dispersal. Switchgrass (*Panicum virgatum* L.) pollen, for example, can travel as far as 3.5 km^50^. In addition to potential long-distance pollen dispersal, the long and precociously exserted stigma of johnsongrass, combined with its xenogamous breeding system, open panicles, and extended flowering periodicity increases the opportunities for outcrossing^25^. Although reports suggest that PMGF is a natural phenomenon in *Sorghum* spp., leading to a wide range of hybridization among members of this genus^15,51–53^, there is a dearth of information regarding intraspecific PMGF in johnsongrass. The current study offers valuable insights in this aspect.

Overall, PMGF in johnsongrass is generally high compared to a number of other agronomically important grass weeds. For example in barnyardgrass, 0.01% PMGF was detected at 50 m in Arkansas^37^, whereas in wild oats (*Avena fatua*), 0.09% PMGF was detected at 56.4 cm distance in Canada^54^. The PMGF frequencies observed between johnsongrass biotypes have significant implications for novel trait movement among *Sorghum* spp. complex in agricultural landscapes. In particular, PMGF can contribute to the spread of herbicide resistance genes among field populations, with significant agronomic implications^55^.

In addition to the breeding compatibility between pollen donor and recipient plants, the frequency of PMGF also depends on pollen density and size of the recipient population^56^. Pollen density is greatly influenced by the size of the pollen donor block^57^; large pollen source fields can lead to high levels of gene flow at greater distances^58–60^. In the current study, both the pollen donor and recipient populations were at the small-plot experimental scale. Though the actual PMGF levels in large fields might vary due to scale-dependent effects, results from this research provide a baseline estimate.

The distance of PMGF is also influenced by the dispersal vector. While johnsongrass is predominantly an anemophilous species, entomophilous pollination especially by Dipteran (*Isomyia paurogonita* Fang & Fan.) and Hymenopteran insects (*Hylaeus* sp., *Sphecodes ephippius* L., *Megachile* sp.) have also been reported^25^. Insect pollination can lead to very long-distance gene flow, depending on the foraging behavior of the insect vector^61^. However, the activity of insect pollinators and their contribution to PMGF were not documented in this study.

### Influence of environmental parameters

**Figure 6:**
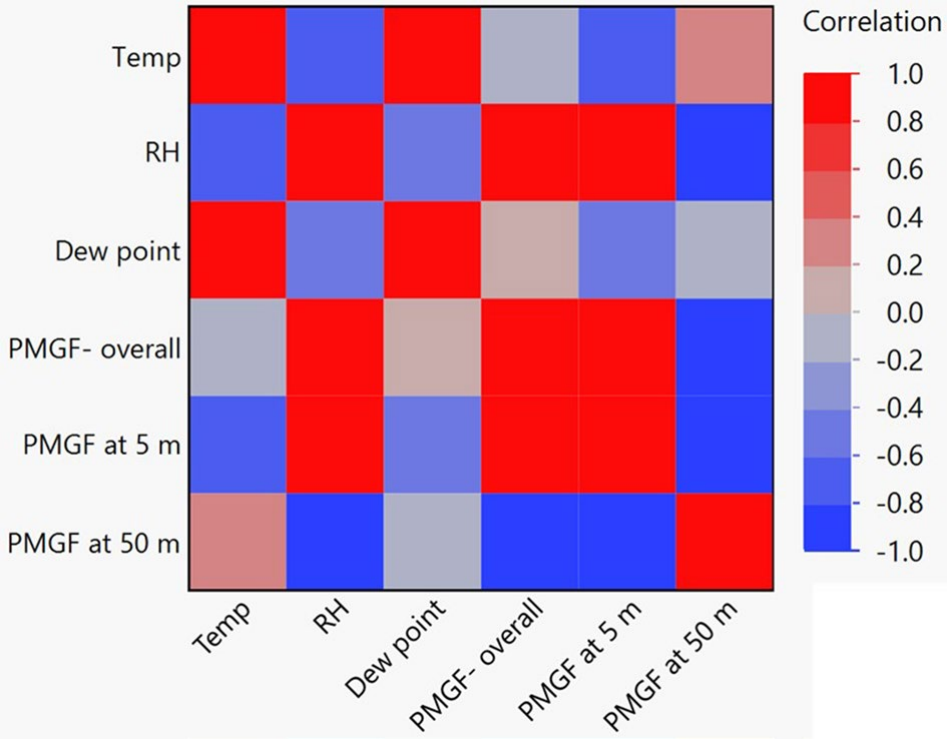
Correlation map showing the relationship between gene flow and the environmental parameters in field studies conducted across three environments: Environment 1, Jun–July, 2018; Environment 2, October–November, 2018; and Environment 3, August–September, 2019. *Temp* temperature, *RH* relative humidity (%), *PMGF* pollen mediated gene flow (%).

The effects of environmental conditions, especially temperature, relative humidity, and dew point, on PMGF were evident in the current study. The ENV1 (summer environment) generally had lower average PMGF values compared to ENV2 and ENV3 (fall environments). PMGF within short distance (i.e. 5 m) was negatively correlated with temperature (r = 0.7, p < 0.05) (Fig. 6), but positively with humidity (r = 0.9, p < 0.001). The temperatures were generally cooler and humidity levels were greater in ENV2 and ENV3, with high levels of corresponding PMGF frequencies. Pollen and stigma, the two indispensable reproductive organs involved in pollination, are highly sensitive to environmental stressors such as hot and dry weather conditions^62,63^, likely explaining the relatively low average PMGF rates in ENV1. At the greater distance of 50 m, PMGF showed a negative correlation with RH and a positive correlation with temperature. High relative humidity typically increases the weight of pollen grains and thereby reduces the distance of pollen transport through wind^64^.

**Figure 7:**
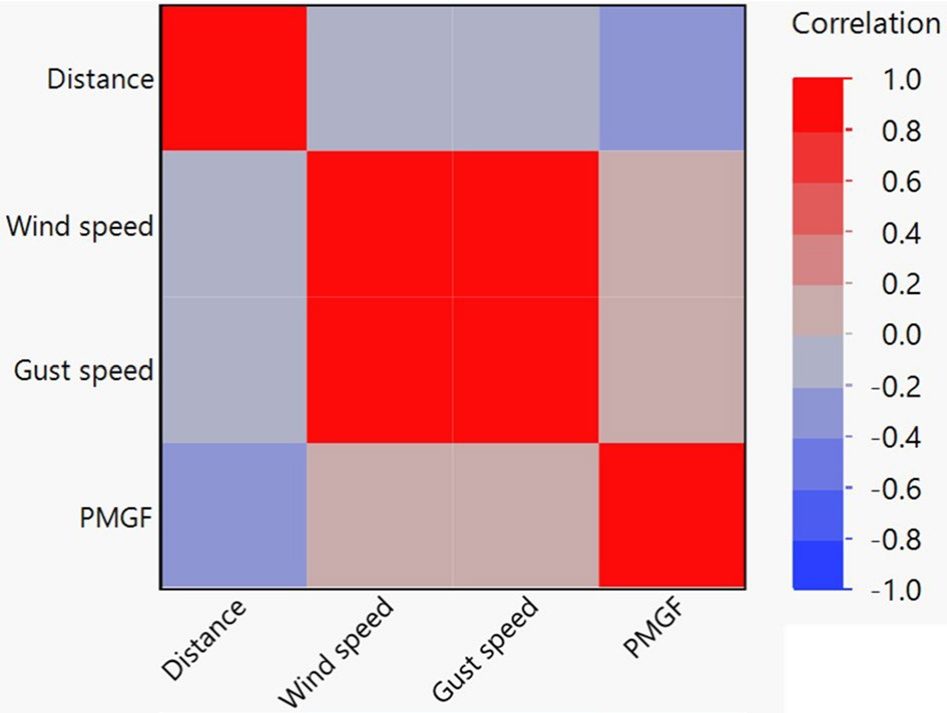
Map showing correlation among PMGF, distance, and the wind parameters (wind speed, gust speed) in field gene flow experiments conducted across three environments: Environment 1, Jun–July, 2018; Environment 2, October–November, 2018; and Environment 3, August–September, 2019. *PMGF* pollen mediated gene flow.

The wind parameters, especially the frequency of wind in a given direction did not show a consistent association with PMGF (Fig. 4). Although PMGF was strongly correlated with distance from the pollen source and pollen was predominantly dispersed by wind, correlation analysis failed to provide a strong association between PMGF and wind parameters (Fig. 7). For example, at the 5 m distance in ENV1, PMGF in the E direction appeared to be consistent with the wind frequency, whereas in the W and NW directions wind flow was infrequent, yet recorded the highest PMGF rates (32.3% and 21.3%, respectively). On the other hand, frequent wind flow was observed in the SE direction, but PMGF was relatively low at 3.6% (Fig. 4A,C). Similar observations were also noted in ENV2 and ENV3.

The frequency, speed, and direction of wind flow were highly variable within and among the ENVs (Fig. 4A–C) and so were the wind gusts (data not shown). The most common wind speed was 0–2 m s^−1^ followed by 2–4 m s^−1^ across the ENVs (Fig. 4A–C). Even though wind frequency and speed highly varied across the directions, almost all directions received at least some frequency of wind, which might have moved a considerable amount of pollen, especially at closer distances, but subsequent pollination might have been influenced by several other factors. More importantly, the random wind gusts might have greatly contributed to the lack of association observed here because gust events can move pollen in an unpredictable fashion at different directions and distances. This might explain the high PMGF frequencies observed at 5 m distance in W and NW directions in ENV1 (Fig. 4A,D), though the wind frequency and speed were low.

Sarangi et al.^38^ reported a significant association of wind frequency and speed with gene flow (%) in waterhemp (*Amaranthus tuberculatus*) in Nebraska, as opposed to our findings. However, they used a clean field in their study, where all the plants other than the pollen recipient blocks were killed, which was also the case with Bagavathiannan and Norsworthy^37^ while studying PMGF in barnyardgrass (*Echinochloa crus-galli*) in Arkansas. In the current study, the presence of tall and dense weed vegetation between the pollen donor and recipient blocks might have diffused pollen movement, affecting pollen flow patterns. Thus, PMGF was highly random at greater distances. Moreover, pollen competition is also expected to be high at farther distances due to the high ratio of AS/AR pollen. The impact of self-pollen competition on PMGF has been observed in various plant species^65,66^, including members of the genus *Sorghum*^67,68^. Jhala et al.^69^ reported no effect of wind speed or direction on PMGF in flax in Alberta, Canada, which corroborates our findings.

### Molecular confirmation of hybrids

**Figure 8:**
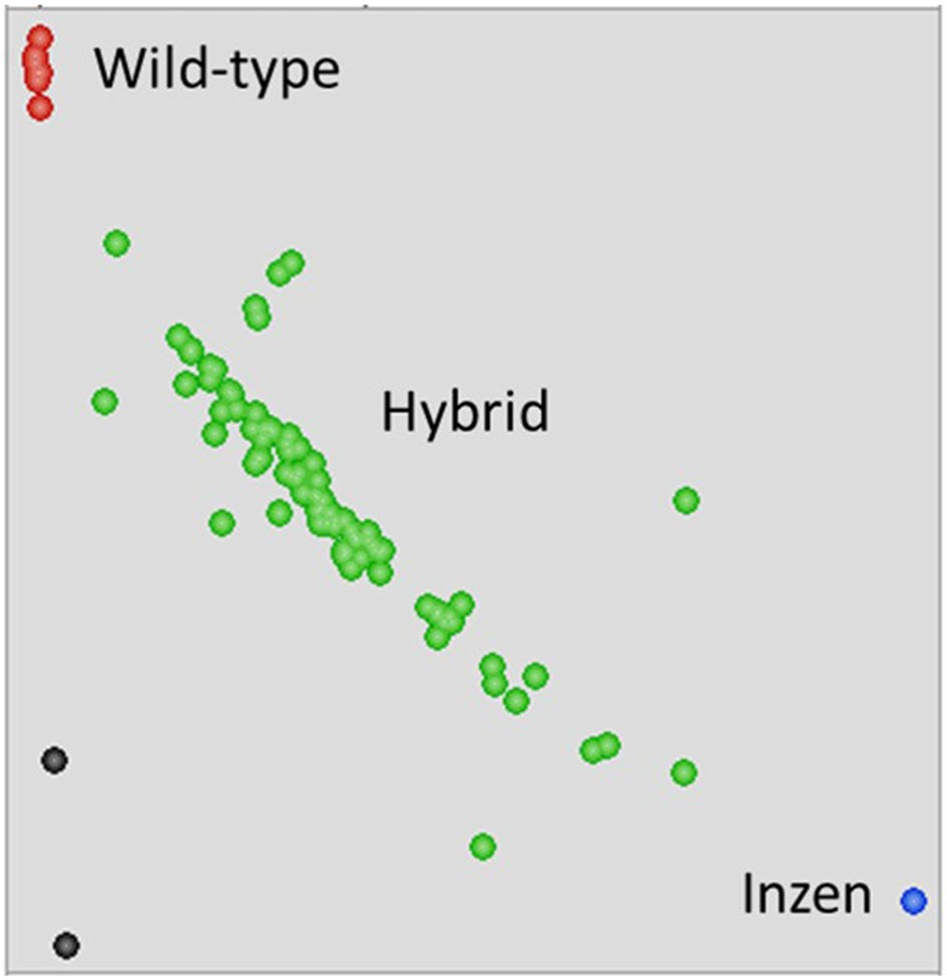
A scatterplot showing fluorescence indices of Fluorescein Amidite dye (FAM) (Y-axis) and Hexachloro-fluorescein dye (HEX) (X-axis) fluorophores for hybrid progenies (90 samples), ALS-inhibitor-resistant johnsongrass (2 samples), wild-type johnsongrass (1 sample) and ALS-inhibitor-resistant (Inzen) sorghum (1 sample).

The hybrids were first confirmed phenotypically based on survival to the discriminant dose of nicosulfuron; the susceptible plants were dead within 14–21 days, whereas the resistant ones survived the application (Fig. 3). Survival to herbicide is a convenient assay for scoring PMGF in this case. However, the molecular investigation conducted on randomly selected plants that survived the herbicide application served as a confirmative test. The Trp_574_Leu mutation, which is present in the AR plants and confers ALS-inhibitor resistance, was detected in the plants determined to be hybrid progenies (Fig. 8). The molecular detection of the Trp_574_Leu mutation was a straight-forward process, and provided definitive confirmation of the hybrid progeny.

## Conclusions

Results of this study offer a baseline estimate of PMGF in johnsongrass. Significant levels of PMGF occurred from AR to AS johnsongrass biotypes under the experimental conditions in southeast Texas. PMGF was detected even at a 50 m distance from the resistant pollen source, which was the farthest distance studied here. However, findings suggest that gene flow in this species could occur for much farther distances, though the frequencies are expected to be much lower. Several factors such as the size of pollen-donor and recipient populations, the speed and direction of wind gusts, temperature, and humidity can greatly influence the degree of PMGF under production field conditions. This is the first study of its kind that demonstrates the significance of PMGF for the transfer of herbicide resistance between field populations of johnsongrass. Thus, field management programs should consider eliminating opportunities for PMGF through various agronomic management approaches. In this regard, best management practices should include management of late-season (flowering) johnsongrass as well as field-edge and roadside populations. Future research should include a more elaborate investigation of PMGF in johnsongrass under production-scale field conditions.

## Supplementary Information

Supplementary Table S1.

## Data Availability

All data used in the manuscript can be accessed in Dryad Data Repository (will be submitted upon formal acceptance of the manuscript).

## Abbreviations

ACCase: Acetyl coenzyme-A carboxylase
ALS: Acetolactate synthase
EPSPS: 5-Enolpyruvylshikimate-3-phosphate synthase
PMGF: Pollen-mediated gene flow
AR: ALS-inhibitor resistant
AS: ALS-inhibitor susceptible
