# Supplementary material for: Pollen-mediated transfer of herbicide resistance between johnsongrass (Sorghum halepense) biotypes
Source: Sci Rep. 2022 May 10;12:7663. doi: 10.1038/s41598-022-11713-8 (PMC9091218; doi:10.1038/s41598-022-11713-8)
Supplement: Supplementary file 1 — Supplementary Table S1. [file 41598_2022_11713_MOESM1_ESM.docx]

**Supplementary Table S1**. Frequency of gene flow in johnsongrass for distances up to 50 m from the pollen source in the four cardinal and four ordinal directions across three study environments*

| Distance | Directions from the pollen donor block (ALS-inhibitor resistant plants) | | | | | | | | |
| --- | --- | --- | --- | --- | --- | --- | --- | --- | --- |
|  | N | NE | E | SE | S | SW | W | NW | Average |
|  | *Environment 1: Gene Flow (%)* | | | | | | | | |
| 5 m | 2.4 | 2.2 | 8.1 | 3.6 | 5.0 | 1.8 | 32.3 | 21.3 | 9.6 |
| 10 m | 2.0 | 2.3 | 6.3 | 0.0 | 6.0 | 1.1 | 0.0 | 3.6 | 2.7 |
| 15 m | 4.3 | 1.4 | 3.1 | 1.8 | 2.2 | 1.2 | 9.1 | 1.3 | 3.1 |
| 20 m | 2.1 | 2.2 | 2.9 | 3.2 | 2.8 | 0.3 | 0.6 | 3.1 | 2.1 |
| 25 m | 2.6 | 3.5 | 4.7 | 0.9 | 6.0 | 1.1 | 0.0 | 0.0 | 2.3 |
| 30 m | 0.0 | 0.4 | 2.5 | 1.9 | 2.8 | 1.1 | 0.3 | 2.6 | 1.4 |
| 35 m | 2.0 | 1.4 | 0.0 | 1.7 | 0.9 | 3.1 | 5.6 | 5.7 | 2.6 |
| 40 m | 2.7 | 0.8 | 2.4 | 6.2 | 4.6 | 1.7 | 0.2 | 0.6 | 2.4 |
| 45 m | 1.0 | 1.8 | 7.1 | 0.0 | 4.4 | 0.0 | 0.8 | 0.0 | 1.9 |
| 50 m | 0.0 | 0.5 | 3.5 | 1.5 | 1.3 | 1.7 | 1.4 | 0.0 | 1.2 |
| Average | 1.9 | 1.7 | 4.1 | 2.1 | 3.6 | 1.3 | 5.0 | 3.8 |  |
| *Environment 2: Gene Flow (%)* | | | | | | | | | |
| 5 m | 2.7 | 0.0 | 2.5 | 15.4 | 45.0 | 8.3 | 15.2 | 40.8 | 16.2 |
| 10 m | 0.8 | 0.0 | 1.5 | 6.5 | 0.0 | 4.2 | 3.4 | 4.5 | 2.6 |
| 15 m | 2.4 | 0.0 | 0.0 | 2.7 | 0.0 | 0.0 | 0.0 | 7.1 | 1.5 |
| 20 m | 3.7 | 0.0 | 0.0 | 0.0 | 2.4 | 0.0 | 3.2 | 1.3 | 1.3 |
| 25 m | 0.0 | 0.0 | 7.9 | 11.4 | 0.0 | 1.9 | 0.0 | 0.0 | 2.7 |
| 30 m | 1.2 | 3.6 | 12.2 | 3.6 | 7.4 | 4.2 | 0.0 | 0.0 | 4.0 |
| 35 m | 4.3 | 0.0 | 2.9 | 3.3 | 10.5 | 1.2 | 0.0 | 3.3 | 3.2 |
| 40 m | 0.0 | 0.0 | 3.9 | 0.7 | 0.0 | 0.0 | 3.8 | 0.0 | 1.1 |
| 45 m | 0.0 | 0.0 | 0.0 | 2.2 | 3.0 | 2.8 | 0.0 | 0.0 | 1.0 |
| 50 m | 5.1 | 0.0 | 1.4 | 0.0 | 0.0 | 0.0 | 0.0 | 0.0 | 0.8 |
| Average | 2.0 | 0.4 | 3.2 | 4.6 | 6.8 | 2.2 | 2.6 | 5.7 |  |
| *Environment 3: Gene Flow (%)* | | | | | | | | | |
| 5 m | 6.4 | 11.7 | 7.5 | 4.3 | 0.0 | 34.0 | 12.5 | 39.7 | 14.5 |
| 10 m | 0.0 | 0.0 | 1.2 | 0.0 | 2.1 | 20.4 | 0.0 | 0.0 | 3.0 |
| 15 m | 0.0 | 10.9 | 0.0 | 0.0 | 0.0 | 2.4 | 0.0 | 0.0 | 1.7 |
| 20 m | 0.0 | 0.0 | 0.0 | 0.0 | 0.0 | 0.0 | 0.0 | 0.0 | 0.0 |
| 25 m | 2.2 | 0.0 | 2.5 | 0.0 | 9.3 | 0.0 | 0.0 | 0.0 | 2.0 |
| 30 m | 0.0 | 0.0 | 2.2 | 0.0 | 9.1 | 0.0 | 0.0 | 0.0 | 1.6 |
| 35 m | 5.4 | 0.0 | 0.0 | 7.9 | 0.0 | 7.2 | 0.0 | 0.0 | 3.4 |
| 40 m | 0.0 | 0.0 | 0.0 | 12.5 | 0.0 | 0.0 | 0.0 | 0.0 | 2.1 |
| 45 m | 2.1 | 4.4 | 0.0 | 0.0 | 0.0 | 0.0 | 0.0 | 0.0 | 0.9 |
| 50 m | 0.0 | 8.7 | 0.0 | 0.0 | 0.0 | 0.0 | 0.0 | 0.0 | 1.1 |
| Average | 1.6 | 4.5 | 1.5 | 2.5 | 2.6 | 6.4 | 1.4 | 4.4 |  |

*Johnsongrass seed collection time: Environment 1, July 2018; Environment 2, November 2018; and Environment 3, September 2019; Abbreviation: ALS – acetolactate synthase
